# GENDER DIFFERENCES IN THE EFFECT OF DIFFERENT EXERCISE DOSES ON PREVENTING DEPRESSIVE SYMPTOMS IN OLDER ADULTS

**DOI:** 10.1093/geroni/igz038.610

**Published:** 2019-11-08

**Authors:** Mei-Lan Chen, Yu-Chen Chang, Ying-Yu Chao, Douglas S Gardenhire, Elisabeth O Burgess

**Affiliations:** 1 Byrdine F. Lewis College of Nursing and Health Professions, Georgia State University, Atlanta, Georgia, United States; 2 Department of Community Health, Chia-Yi Christian Hospital, Chiayi, Taiwan; 3 School of Nursing, Rutgers, the State University of New Jersey, Newark, New Jersey, United States; 4 Gerontology Institute, Georgia State University, Atlanta, Georgia, United States

## Abstract

Regular exercise is potentially an effective way to prevent or reduce depressive symptoms for older adults. However, little research has focused on gender differences in exercise dose effects on depressive symptoms in older people. The purpose of this study was to test gender differences in the preventive effects of different exercise doses on depressive symptoms among community-dwelling older adults in Taiwan. This study was a secondary analysis of a longitudinal cohort study in a sample of older Taiwanese adults (N = 2,673; mean age 74.2 ± 5.7 years). Four different doses of moderate-intensity exercise were examined including three 15-min sessions/week, three 30-min sessions/week, six 15-min sessions/week, and six 30-min sessions/week. Descriptive statistics and generalized linear mixed models were used to analyze characteristics of the sample and hypotheses testing. All analysis models were adjusted according to age, gender, education, marital status, smoking, social participation, and chronic conditions. The results indicated that regular exercise with at least 15 min per session, 3 times a week of moderate intensity was significantly associated with lower levels of depressive symptoms for women, but there were no significant preventive effects on depressive symptoms for men. This study suggests that moderate-intensity exercise may play a protective role in depression prevention for older women, even with a very low dose (three 15-min sessions/week). Gender differences should be considered for future research and clinical practice when designing exercise interventions on preventing depression for older adults.

